# Mosquito control via inbuilt net hoisting windows: the inverted S/O channel/grip device option

**DOI:** 10.5281/zenodo.10876543

**Published:** 2015-12-09

**Authors:** Francis S.O. Ugwu

**Affiliations:** 1 South East Zonal Biotechnology Centre, University of Nigeria, Nsukka, Nigeria.

## Abstract

**Background:**

In many tropical countries, malaria remains a major health problem. Effective control of in -house mosquitoes, due to entry prevention, takes advantage of the behavioural preferences of endophagy and endophily of mosquitoes. Insecticide-treated nets (ITN) adopt this, but is burdened with challenges, which result in dwindling adoption of the methodology in the tropics. An alternative is prevention of vector-human contact through house modifications, which adopts S/O channel/grip devices that form attachments to existing windows. Inverted S/O channel/grip frame (ISOWF) was therefore devised as an integrated window frame, which could be used for direct net attachment and housing shutters. The ISOWF is a lightweight material weighing 1/6 of the equivalent size of wood.

**Materials and methods:**

Appropriate dies were employed to form channels from thin iron sheets in the prefer red shape and size of a laterally inverted letter ‘S’ (ƨ). The upper half was minimised to form and facilitate the ‘O’ griping of nets, while the remaining half was bloated to house window shutters. Net hoisting or de-hoisting periods were determined by timing. A room screened with a net was attached to the device and situated next to a mosquito breeding room. The room was charged with adequate carbon dioxide attractant for mosquitoes and protected with ISOWF screen. This was used to evaluate the effectiveness of the method. The time taken to hoist the net was measured. The frame was constructed like a metal/ wooden frame, except that the anterior view had two steps.

**Results:**

The average time taken to hoist or de-hoist a net across a 60 cm x 120 cm window, formed by the frame, was 5.96 and 1.68 minutes, respectively. The nets retained their integrity. Mixed mosquito populations numbering 1,341 in total could not gain access to a room with carbon dioxide attractant, and the ISOWF screen prevented passage.

**Conclusion:**

The ISOWF acts as a potential mosquito entry-prevention device, which further provides reinforcement to house screening. It forms an effective mosquito control device, which brands house screening as a sustainable environment for mosquito control, and subsequently, malaria control. This will also control the overall indoor densities of nuisance mosquitoes and other insect vectors.

## 1 Introduction

Malaria remains a major health problem in many tropical countries [1]. Currently, the most promoted means of reducing human-mosquito vector contact is through the use of bednets and, to a lesser extent, mosquito repellents [2,3]. Insecticide-treated nets (ITN) reduce malaria, particularly in young children and pregnant women [4]. There is convincing evidence that its pervasive and widespread application has significantly impacted malaria morbidity and mortality across Africa. The encouragement of increased ITN distribution and funding has therefore been justified [4-7]. Seidlein *et al.* [8] cited the reduction of bednet usage in two West African countries and also raised concern that the uptake and sustained use of bednets in hot and humid tropical Africa may not be attainable in future if the immediate benefit of bednets in preventing insect bites outweighs the discomfort caused by bednets. It was also shown that large populations at risk of malaria in countries like Nigeria continue to have low coverage of bednets [8,9].

Mosquito control activities at community level can utilise approaches that directly reduce human-mosquito contact, as well as methods that reduce the total number of mosquitoes in the environment. Bamidele *et al*. [10] indicated that planned community participation to control malaria promoted awareness and motivated people to study the problem, as well as proffer solutions. Such approaches may include larval source management, where mosquito reduction is targeted with chemicals or biological agents. However, application of insecticides and drugs against mosquito vectors and malaria, respectively, are becoming less effective due to resistance [11,12] and altered vector behaviour [4,13]. Their uses entail strict control and require adherence to endorsement by WHO [14]. Moreover, massive indoor residual spraying (IRS) against adult mosquitoes is often subject to policy changes because of safety concerns, low community acceptance, and high cost [3].

A major issue with dependence on ITNs and IRS is the hope that there will always be a potent insecticide that will kill mosquitoes on contact. Unfortunately, the reality is that when a new public health insecticide is introduced it tends to gain popularity and widespread use will inevitably result in resistance to it. Recently, Cohen *et al.* [15] reviewed malaria resurgence and attributed it to weak control programmes, for various reasons, of which resource constraints were dominant over other contributing factors such as the intrinsic potential of malaria transmission, and vector or drug resistance. The authors suggested the need to develop practical solutions to the financial and operational problems to effectively sustain successful malaria control programmes. Thomas *et al.* [12] suggested the development and implementation of an integrated multifacetted approach, which would parallel the strategy of integrated pest management in agriculture.

One of the most effective means of sustaining such control is by taking advantage of the vector behaviour, notably endophagy and endophily, to prevent host-vector contact through insect-proofing of houses [13,14,16-18]. Ogoma *et al.* [17] posited that simple changes in house design could safeguard people against exposure to mosquito bites and malaria transmission. Kirby *et al.* [19] indicated that houses built of concrete walls harboured fewer mosquitoes than mud houses, owing to fewer potential access routes which may be available to vectors. Some architectural modifications were recommended for African homes, for improving airflow within houses and to facilitate better vector control [8]. However, it was not certain that the quality of house construction determined the exclusion of indoor resting mosquitoes. In a xeno-monitoring survey, Howell and Chadee [20] noted that well-constructed houses in Trinidad and Tobago, and in the Dominican Republic, harboured more endophagic mosquitoes than poorly constructed ones. They noted that improvements such as window or door curtains had no effect on reducing the number of mosquitoes resting indoors in both countries. This enigma re-emphasised that only an exclusion method could be relied on to protect houses with certainty.

Kirby *et al.* [21,22] and Ugwu [18] observed that an historical but often-neglected method of preventing mosquito-host contact was the hoisting of window nets in hot tropical climate. Peterson *et al.* [23] noted a meagre 3.1% usage. Where window screens were present, it was observed that there were attachments to an existing window frame or a sliding screen (usually inadvertently left agape), in some aluminium-glass windows. The former method usually employed wooden battens, which were nailed, together with the net, to an existing wooden window frame; although, occasionally, some innovative people used thin strips of metal to replace the wooden battens in strapping nets to window frames. In both cases, the nets are left and gather dust, mites and other allergens, thus making them unsightly and unhealthy. According to Ugwu [18], the situation in wooden window-hoisted net is worse because both the batten and the frame may bend, retract, degenerate or rot, thereby creating gaping spaces, which insect vectors can use as access routes. Therefore, the view of Howell and Chadee [20] that alteration of the domestic environment in novel ways to discourage human-vector contact rates, in order to attain disease reduction, should be pursued with vigour.

Inability to replace ineffective screens and the support structures which show signs of decay could be due to lack of funds, tools, time or skills, which might be required to effect such changes. Such difficulties might be worse in high-rise buildings where, in addition, hiring of tall ladders or erecting scaffolds around the buildings could pose additional problems. These defaults could compromise the

realisation of some of the objectives of the Millennium Development Goals [7]. The identified problems could be solved by the adoption of S/O channel/grip devices [18]. However, the device must be attached to an existing window frame. The main objectives of this current study are to make the hoisting technique more attractive, to simplify the process further and to ensure that the additional burden of attaching extraneous channel hangers on windows is eliminated. Moreover, it aims to simplify net hoisting across windows, while still retaining all the qualities of its predecessor, reported in a previous publication [18].

## 2 Materials and methods

### 2.1 Construction of inverted S/O channel/ grip device

**Figure 1.**
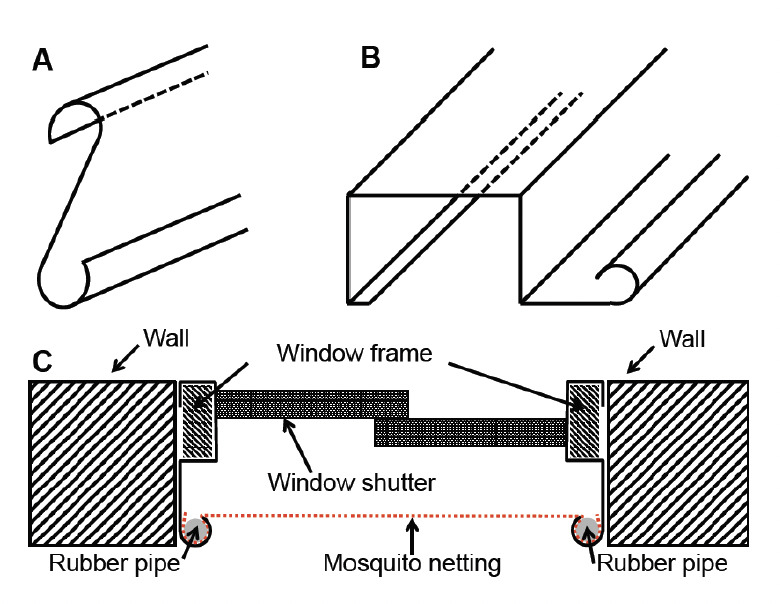
Mosquito net hoisted with inverted S/O channel/ grip window frame. A: Model inverted S channel. B: Piece of sheet metal formed into laterally inverted S/O channel/grip window frame with minimised gripping half for attaching net to it and the bloated remaining half for housing of window shutters. C: Cross-section of inverted S/O channel/grip window frame with shutters and hoisted mosquito netting.

The steps adopted in the construction of S/O channel/grip devices were similar to those described by Ugwu [18]. However, the method was used with some modifications. The difference was that appropriate dies (the inverted S-channel die is a block of metal whose interior has been shaped in form of an inverted ‘S’ (ƨ), such that when a flat shaped metal is pressed into it, it takes up that shape), were employed to obtain channels in the preferred shape and size of a laterally inverted letter ‘S’ (ƨ). The right side of the inverted (ƨ) faced downwards, while the left side faced upwards. The upper half of the channel, which was to be at the distal end of the window of a room to be protected, was minimised, while the remaining half was bloated so as to accommodate the shutters and their housing, see Figure 1). A sheet metal was therefore marked according to the desired dimensions, and holes 15 cm apart were added along the length, which would coincide with the loci where the net would be attached before press treatments (pressing the flat sheet into the die). This part of the channel would also form part of the base of the window frame. The resulting channel was used to form the perimeter of an ‘Alumaco’ window, measuring 60 cm x 120 cm. A piece of net was attached to the minimised part, with an ‘O’-shaped, firm rubber or plastic pipe which had a diameter greater than the internal diameter of the specified end of the channel in such a way that when it is forced in place, it would strap the net tightly in place.

### 2.2 Net hoisting/de-hoisting times of inverted S/O channel/grip devices

A demonstration was given to ten volunteers on how to fix and remove the net on the ISOWF. The channel had a rectangular surface area of 8450 cm^2^ that needed to be covered with netting. This was the same surface area covered previously as in S/O channel grip devices [18]. Ten volunteers were each given pieces of ‘O’ rubber pipes and a piece of net to hoist in place. Each duration taken to hoist and remove the ISOWF was recorded.

### 2.3 Effectiveness of the inverted s channel screening method to prevent mosquito entry

A house with three sealed rooms, the central one being the mosquito breeding room, was experimentally used to test the effectiveness of the novel frame. The breeding room had two connecting window, one each opening to an adjacent room. One of the windows was screened with the inverted ‘S’ channel window frame, while the other had S/O channel/grip device attachment [18] as control. Both windows had the same pore size of net (100 holes/cm^2^) screens. A basin in the central breeding room was filled with mosquito larvae, harvested from *egbaite* (the local name for series of pots which are lined up beside a house to collect run-off rain water from the roof of a house after rainfall), used tires and puddles. Larval floating behaviour was used to increase the number of *A nopheles* larvae in the breeding room [24].

The two experimental rooms had a vent each, covered with net and through which a bottle trap of yeast-generated CO_2_, prepared according to Saitoh *et al.* [25], was placed and replaced every 24 h for 14 days, from the day on which adult emergence was noticed in the breeding room. Also on the day of adult emergence, cotton wool balls soaked in glucose water were introduced into the breeding room and also changed every 24 h.

## 3 Results

### 3.1 Inverted S/O channel/grip device

**Figure 2.**
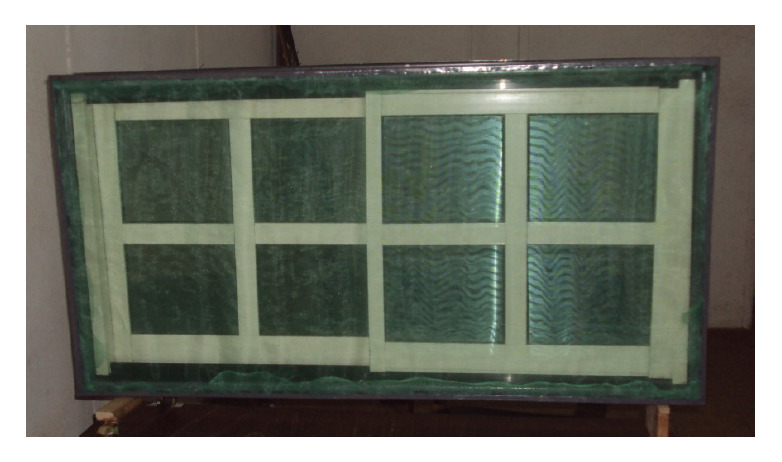
Inverted S/O channel/grip window frame (ISOWF) with hoisted net and housing aluminium window slide shutters (anterior view, note the greenish tinge due to the colour of the mosquito net).

**Figure 3.**
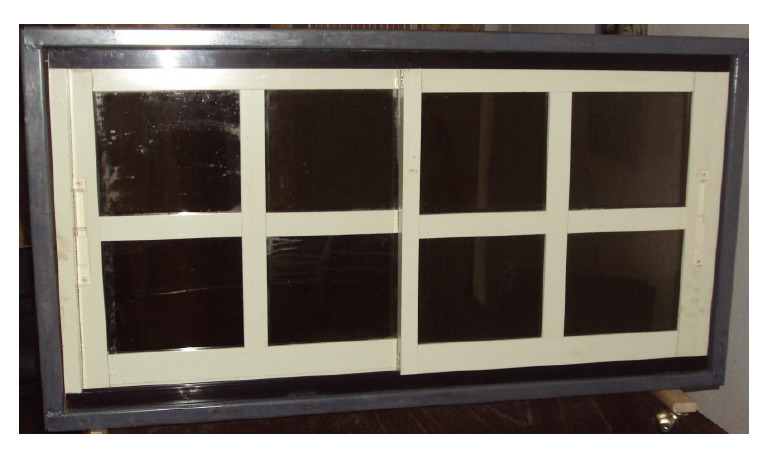
Inverted S/O channel/grip window frame (ISOWF) housing aluminium window slide shutters (posterior view).

The constructed inverted S/O channel/grip window frame was of lightweight material when compared with wood of the same specification. A window frame of the channel, measuring 11 cm width by 240 cm length by 4 cm height, made with thin iron sheet metal of 0.8 mm thickness, weighed approximately 2 kg, whereas the same length of wood (*Chlorophora excelsa,* called *iroko* in the local language), with similar dimensions, weighed six times more. When the channel was cut and joined to form the perimeter of a window space it looked similar to other frames except that the anterior view (the side which would face outside of the room) appeared to be a two-step channel, while the reverse side was like an ordinary wooden or metal window frame. On mounting aluminium-glass slide shutters on the novel frame, it looked attractive. Attaching the screen of polyethylene net, it appeared to reduce the brightness of the aluminium-glass shutters. Also, the anterior side was broadened because the gripping region was stepped down, thereby creating more surface area that the net must cover. Only a narrow rim of channel was conspicuous thereafter, and the excess of the net appeared to blur that edge of the window frame (Figure 2). The posterior view of the mounted window frame is as shown in Figure 3.

### 3.2 Net hoisting/de-hoisting times

Mosquito netting could be hoisted from both the anterior and posterior sides of the channel. The mean hoisting time of the nets was 5.96±0.49 minutes; the mean de-hoisting time was 1.68±0.42 minutes. The ‘step down’ at the gripping end of the channel was impeding the fingers, making fixing the netting less easy. Netting could be removed cleanly each time without any tear or opening.

### 3.3 Effectiveness of the inverted S channel screening method to prevent mosquito entry

The traps were checked daily for mosquitoes. There was no mosquito caught in any of the traps in the experimental or control rooms throughout the duration of the test. At the end of the experiment, all rooms were sprayed with insecticide. There were no mosquitoes seen in either experimental or control rooms. However, 1,341 dead (562 anophelines and 779 aedines) mosquitoes were counted, only in the breeding room. The two channels (the test ISOWF and S/O channel/grip devices) were therefore equally effective means to prevent mosquitoes accessing protected rooms.

## 4 Discussion

It appears that in many parts of Sub-Saharan Africa protective ‘coverage’ in malaria control is almost entirely restricted to bednet deployment, that is, control of mosquitoes within a small sleeping area within a household offering personal protection. It is gratifying that Ogoma *et al.* [17] applied the term to also include house screening. In this research, the challenge was to discourage indoor exposure to vectors [16] and to broaden ‘coverage’ by house modification, to include preventing the entry of mosquitoes via windows, into the entire household that might encompass tens, or even as many as hundreds of bed spaces (depending on the size of the house and the number of windows present), by enhancing hoisting of net screens across windows. The task was accomplished by designing a window frame that allows direct hanging of nets to it without further attachments such as S/O channel/grip devices or the tedious traditional use of wooden battens fastened to window frames. Thus, the novel channel is well positioned to tackle the constraints to house screening [18, 22] and could improve house screening, which seems to have been ‘forgotten’ as a malaria control measure [11]. The net hanging window frame could also facilitate reduction of indoor biting densities of other mosquito species which transmit lymphatic filariasis, several arboviruses, such as Chikungunya, O’Nyong nyong, Rift Valley Fever (RVF) and West Nile Virus [26,27]. When applied to animal houses, it could also prevent zoonoses by preventing human-animal or animal-human transmission of pathogens.

The ISOWF permits a person to hoist the net from both the anterior and posterior sides. The implication is that one could place the net when inside or outside of the room, although when used in a high-rise building, it would be more convenient to do so from inside the room like its forbearer. It was noted that hoisting/de-hoisting periods were longer in ISOWF when compared with its predecessor model [18]. These periods could be reduced if the ‘neck’ (space before) of the ‘O’ grip section is increased to allow more room for manipulation by fingers. However, the functionality of the novel frame was also confirmed as an effective mosquito control approach.

The present work could compose a permanent feature of buildings that would not have the shortcomings of wood, as indicated earlier [18]. Although conceived for new buildings, it could also be applied to existing buildings if desired since this would not require redesigning of the entire structure or parts of the building. Instead, the only necessary change would be the window frame, which may have to be replaced. If a 1.5 m x 1.5 m metal/wooden window frame were in place, the same size of the inverted channel would be required for replacement. The dampening of the usual aesthetic appeal of aluminium-glass window (as in the experimental window – see Figure 2) was inevitable because insects will be screened out. The loss of window brightness could be mitigated by using netting materials of brighter colours. The excess of netting materials at points of attachment could be avoided by using precise net measurements before cutting (the netting material shown in Figure 2 is intentionally superfluous).

When comparing ITN usage with no net usage in Africa, Gamble *et al.* [28] found that mean birth weight increased by 55 g, while low birth weight and miscarriages/ stillbirths was reduced by 23% and 33%, respectively, especially in primigravids. Using mathematical modelling, Killeen *et al.* [29] predict that where 50 % of all adults and children are covered by ITNs, they could achieve equitable community-wide benefits equivalent to or greater than personal protection (which could have been obtained only when 80% of the target group was protected). It was therefore concluded that coverage of the entire population would be a precondition to achieve a large reduction in malaria burden. The screening of windows presents a means of achieving that goal, because it forecloses the lack of utilisation peculiar to bednets, when one is available [6]. Kirby *et al.* [19] found sevenfold higher numbers of *Anopheles gambiae s.l.* in rural villages than in urban environments of The Gambia. This was attributed to a number of factors that prevented mosquitoes from entering houses, which were present in an urban setting but absent in villages; the novel device could help to reduce this imbalance. Moreover, Ogoma *et al.* [26] reported that *Culex quinquefasciatus, Cx. univittatus* and *Cx. theileri* mainly prefer windows and doors as their main points of entry, while Okumu *et al.* [30] found that bloodfed mosquitoes preferred to exit through windows; thus, the use of window screens would frustrate the exit of fed vectors and might contribute to a reduction in vector fecundity and concomitant reduction of malaria cases. The application of innovations like the inverted S channel could therefore drastically reduce the number of endophagic mosquitoes in houses.

The inverted S channel was conceived to be applied to windows only, but its application could be extended to include ceilings, eaves and vents, similar to its predecessor [18]. It could also be used for making net tent classrooms [31], and for building tents in general. It could also be employed in experimental houses for speedy installation, greater flexibility and especially for interior walls, such as the Ifakara type [30]. Homes with improved airflow had been envisioned for Africa [8], and the inverted S channel and its predecessors [18,31] can be easily made: denser nets could be removed when airflow becomes unacceptably low and be replaced with nets with bigger mesh sizes. It could enhance the low negative feedback associated with house screening [22]. As vector behavioural changes in favour of outdoor biting has been identified [13], sleeping outdoors should be in net-tents [31] made with either inverted S (ƨ) or S/O channel/grip devices. During displacement of large human and animal populations, their temporary camp and resettlement could be quickly and cheaply constructed with these channels. This study was, however, limited to windows, which open by sliding its shutters on one another or window shutters arranged in panes like glass louvres. Swing-out windows cannot be applied to this model. Moreover, the netting screen should not be regarded as a form of physical protection against falling objects or persons [32].

## 5 Conclusion

The challenge was to broaden ‘coverage’ by house modification to include preventing the entry of mosquitoes via windows. The response to this task resulted in the inverted s/o channel window frame, which allows for direct hanging of nets without further attachments. Screening with this novel channel is well positioned to tackle the constraints to house screening and could replace bednets as primary malaria control measure. The net hanging window frame could also reduce indoor biting densities of other nuisance and vector mosquitoes.
